# Supplementary material for: Physically Motivated Improvements of Variational Quantum Eigensolvers
Source: J Chem Theory Comput. 2024 Jun 10;20(12):5133–44. doi: 10.1021/acs.jctc.4c00329 (PMC11209943; doi:10.1021/acs.jctc.4c00329)
Supplement: Supplementary file 1 — ct4c00329_si_001.pdf [file ct4c00329_si_001.pdf]

Supporting Information: Physically motivated improvements of Variational Quantum Eigensolvers

Nonia Vaquero-Sabater,[†,‡] Abel Carreras,[¶] Román Orús,[†,¶,§] Nicholas J. Mayhall,[‖] and David Casanova*,[†,§]

†*Donostia International Physics Center (DIPC), 20018 Donostia, Euskadi, Spain*

‡*Polimero eta Material Aurreratuak: Fisika, Kimika eta Teknologia Saila, Kimika Fakultatea, Euskal Herriko Unibertsitatea (UPV/EHU), PK 1072, 20080 Donostia, Euskadi, Spain.*

¶*Multiverse Computing, 20014 Donostia, Euskadi, Spain*

§*IKERBASQUE, Basque Foundation for Science, 48009 Bilbao, Euskadi, Spain*

‖*Department ofChemistry, Virginia Tech, Blacksburg, Virginia 24061, USA*

E-mail: david.casanova@dipc.org

Contents

1 Orbital symmetry **S2**

2 Fidelity of the density matrix **S3**

3 Dependence with active space **S4**

4 Energy measurements **S5**

5 Circuit depth **S6**

6 FCI energy curves S7

1 Orbital symmetry

Unrestricted Hartree-Fock (UHF) allows for the molecular orbitals to break the symmetry between spin-α and spin-β orbitals, that is, different spatial functions for spin-up and spin-down electrons. This flexibility triggers the possibility to lower the HF energy in the presence of strong electron correlations, e.g., stretched bonds. But this addition energy minimization implies an unphysical lose of symmetry (Figures S1a and S2a). As a consequence, the final wavefunction is not an eigenstate of $\hat{S}^2$ (spin contaminated wavefunction).

On the other hand, natural orbitals (NOs) obtained by the diagonalization of the total UHF electron density matrix ($\rho = \rho_\alpha + \rho_\beta$) recover the symmetry between α and β electrons (Figures S1b and S2b). Moreover, NOs with fractional occupancies correspond to symmetry irreducible representations of the Hamiltonian symmetry point group. Notice that this not necessarily the case within degenerate orbital spaces (occupancy degeneracy), e.g., in fully unoccupied orbitals.

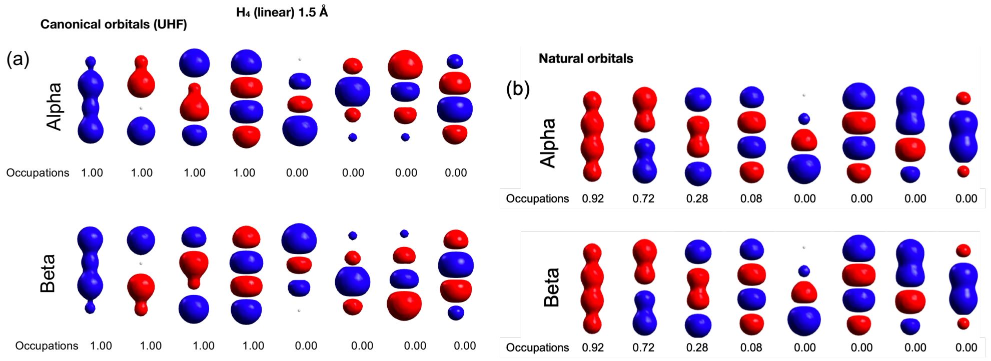

Figure S1: UHF canonical (a) and natural (b) orbitals and their occupancies for the H_4 system with H−H distance of 1.5 Å.

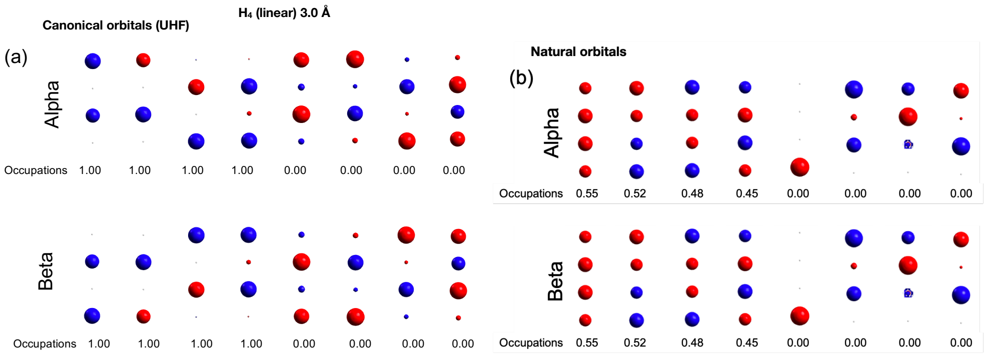

Figure S2: UHF canonical (a) and natural (b) orbitals and their occupancies for the H_4 system with H−H distance of 3.0 Å.

2 Fidelity of the density matrix

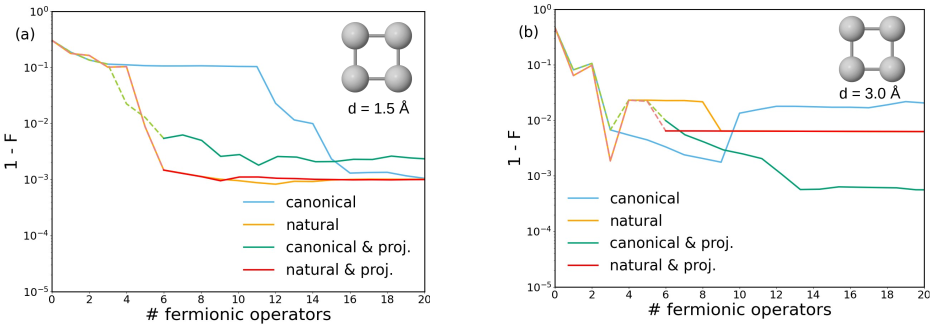

Figure S3: Fidelity of ADAPT-VQE wavefunctions with respect to FCI obtained for the square H_4 system with H−H distance of 1.5 Å (c) and 3.0 Å (d), performed with canonical (blue), natural (orange), projected canonical (green), and projected natural (red) orbitals. Initial iterations with orbital subspaces indicated with dashed lines.

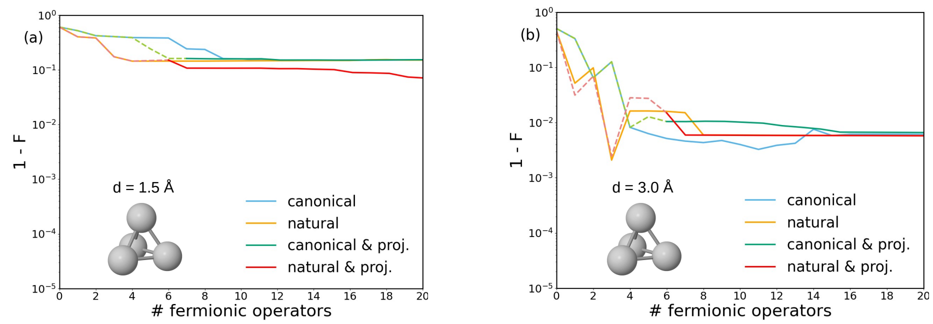

Figure S4: Fidelity of ADAPT-VQE wavefunctions with respect to FCI obtained for the tetrahedral H_4 system with H – H distance of 1.5 Å (c) and 3.0 Å (d), performed with canonical (blue), natural (orange), projected canonical (green), and projected natural (red) orbitals. Initial iterations with orbital subspaces indicated with dashed lines.

3 Dependence with active space

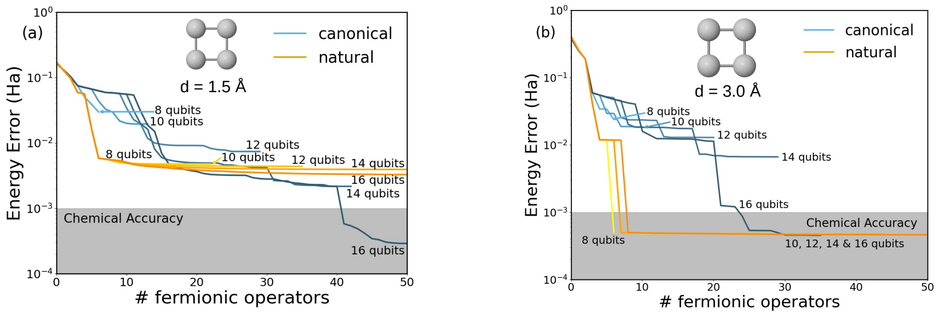

Figure S5: Energy error (in Hartree) with respect to FCI of ADAPT-VQE obtained with standard orbitals (blue) and NOs (orange) with different number of active spaces (from 4 to 8 orbitals) for the square H_4 with 1.5 Å (a) and 3.0 Å (b) H – H distances, and computed with the 3-21G basis set.

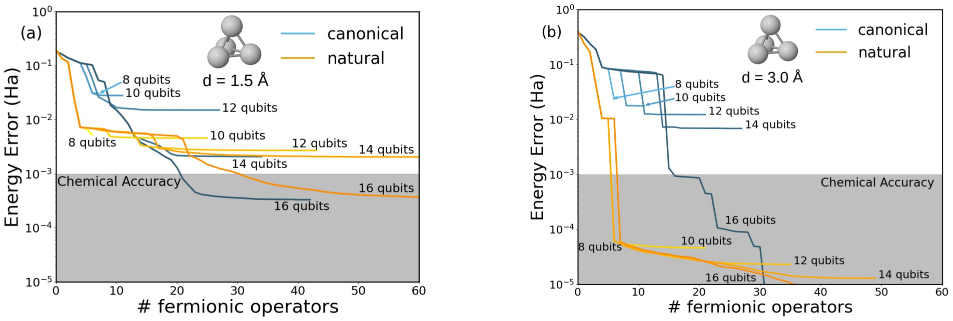

Figure S6: Energy error (in Hartree) with respect to FCI of ADAPT-VQE obtained with standard orbitals (blue) and NOs (orange) with different number of active spaces (from 4 to 8 orbitals) for the tetrahedral H_4 with 1.5 Å (a) and 3.0 Å (b) H − H distances, and computed with the 3-21G basis set.

4 Energy measurements

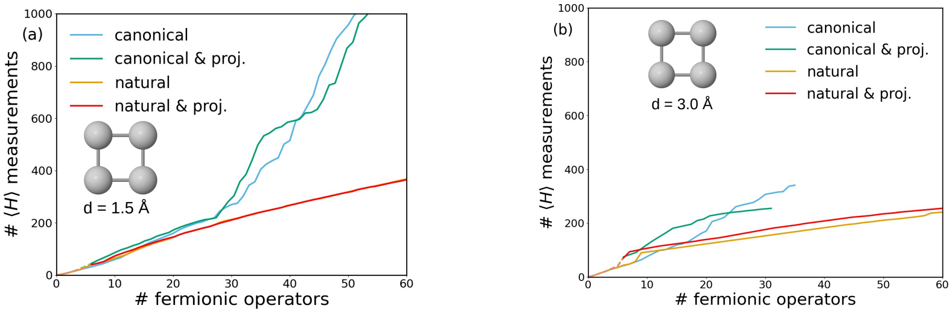

Figure S7: Number of energy measurements ($\langle H \rangle$) for the simulation of the square H_4 system with 1.5 Å (a) and 3.0 Å (b) H − H distances performed with canonical (blue), natural (orange), projected canonical (green), and projected natural (red) orbitals. Initial iterations with orbital subspaces indicated with dashed lines.

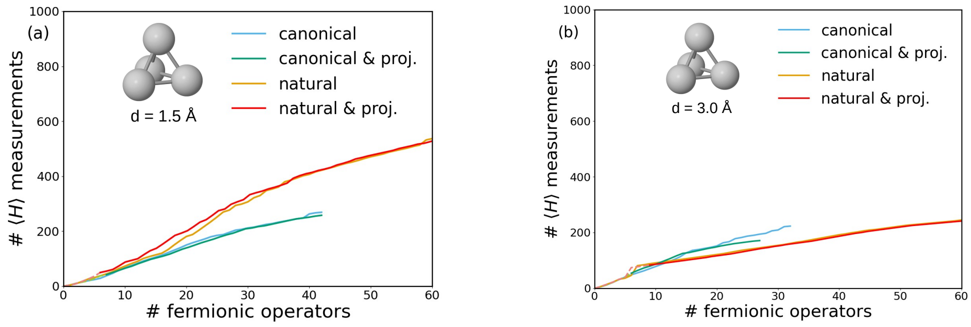

Figure S8: Number of energy measurements ($\langle H \rangle$) for the simulation of the tetrahedral H_4 system with 1.5 Å (a) and 3.0 Å (b) H−H distances performed with canonical (blue), natural (orange), projected canonical (green), and projected natural (red) orbitals. Initial iterations with orbital subspaces indicated with dashed lines.

5 Circuit depth

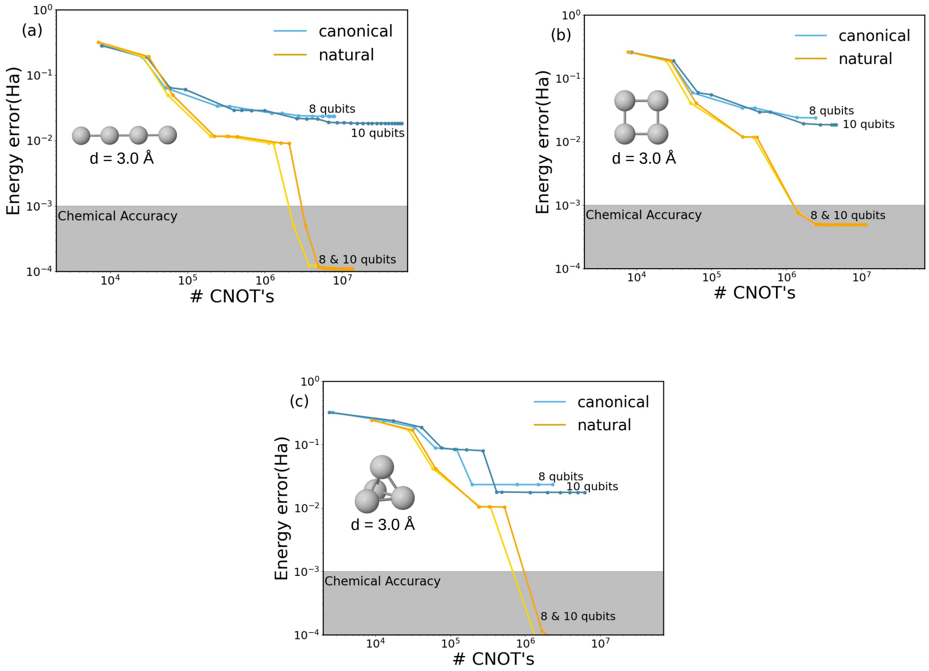

Figure S9: Energy errors (in Hartree) as a function of the number of CNOT gates, calculated for the linear (a), square (b) and tetrahedral (c) H_4 models with $d = 3.0$ Å, using canonical (blue) and natural (orange) orbitals, and with two active spaces (4 and 5 orbitals).

6 FCI energy curves

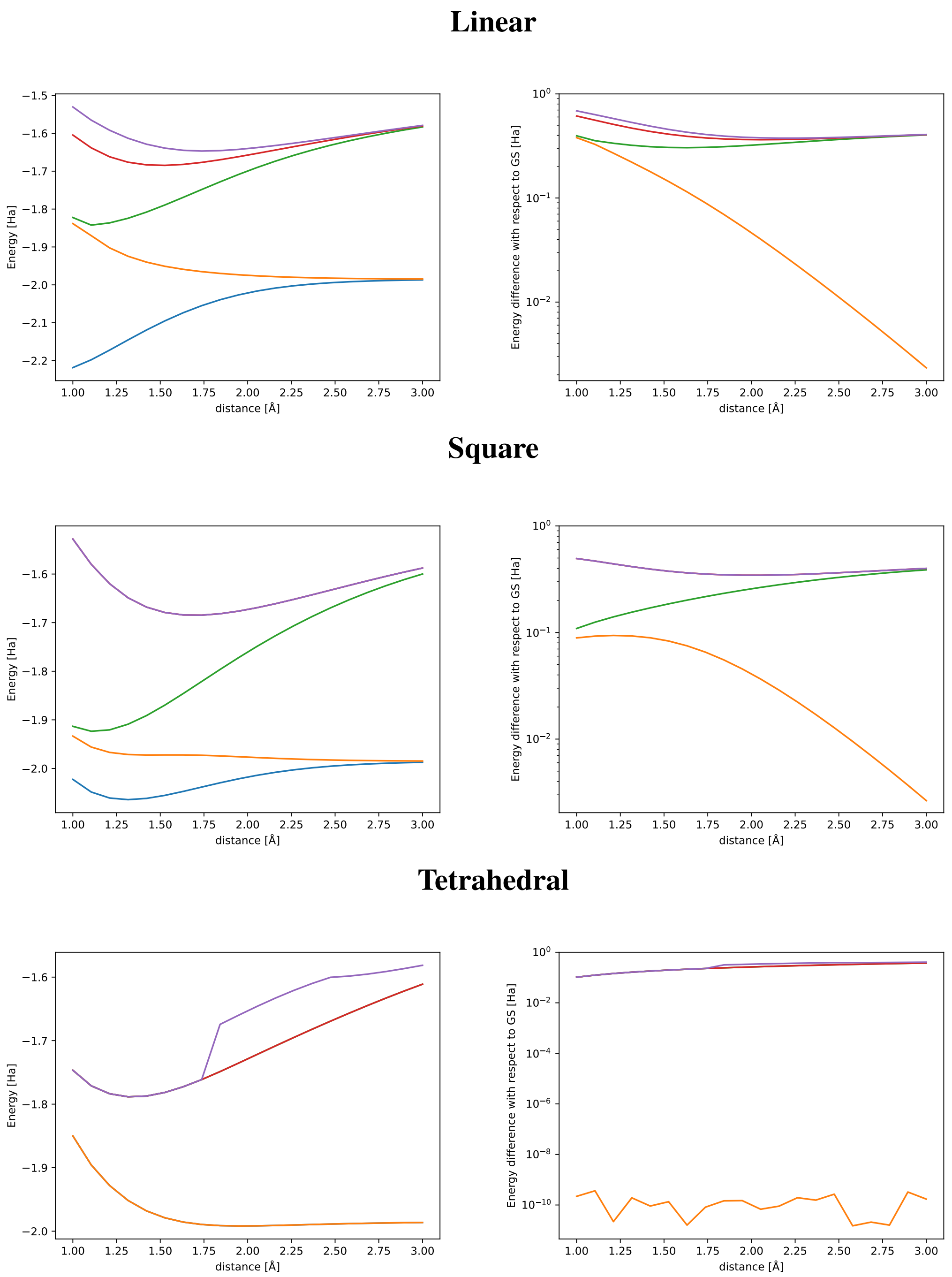

Figure S10: FCI energy curves as a function of the bond distance for the singlet excited states of the studied H_4 molecules. Absolute state energies are shown on the left and energy differences with respect to the FCI ground state on the right.
